# Detection of Alpha-Fetoprotein in Hepatocellular Carcinoma Patient Plasma with Graphene Field-Effect Transistor

**DOI:** 10.3390/s18114032

**Published:** 2018-11-19

**Authors:** Dae Hoon Kim, Hong Gi Oh, Woo Hwan Park, Dong Cheol Jeon, Ki Moo Lim, Hyung Jin Kim, Byoung Kuk Jang, Kwang Soup Song

**Affiliations:** 1 Department of Medical IT Convergence Engineering, Kumoh National Institute of Technology, Gumi, Gyeongbuk 39177, Korea; dhoonkim@kumoh.ac.kr (D.H.K.); oh558@naver.com (H.G.O.); parkwh123@gmail.com (W.H.P.); Vcaptin@kumoh.ac.kr (D.C.J.); kmlim@kumoh.ac.kr (K.M.L.); 2 Biomedical IT Convergence Center, Gumi Electronics and Information Technology Research Institute, Gumi, Gyeongbuk 39171, Korea; hjkim745@geri.re.kr; 3 Department of Internal Medicine, Keimyung University School of Medicine, Daegu 41931, Korea; jangha106@gmail.com

**Keywords:** hepatocellular carcinoma, alpha-fetoprotein, biosensor, graphene, field-effect transistor

## Abstract

The detection of alpha-fetoprotein (AFP) in plasma is important in the diagnosis of hepatocellular carcinoma (HCC) in humans. We developed a biosensor to detect AFP in HCC patient plasma and in a phosphate buffer saline (PBS) solution using a graphene field-effect transistor (G-FET). The G-FET was functionalized with 1-pyrenebutyric acid *N*-hydroxysuccinimide ester (PBASE) for immobilization of an anti-AFP antibody. AFP was detected by assessing the shift in the voltage of the Dirac point (ΔV_Dirac_) after binding of AFP to the anti-AFP-immobilized G-FET channel surface. This anti-AFP-immobilized G-FET biosensor was able to detect AFP at a concentration of 0.1 ng mL^−1^ in PBS, and the detection sensitivity was 16.91 mV. In HCC patient plasma, the biosensor was able to detect AFP at a concentration of 12.9 ng mL^−1^, with a detection sensitivity of 5.68 mV. The sensitivity (ΔV_Dirac_) depended on the concentration of AFP in either PBS or HCC patient plasma. These data suggest that G-FET biosensors could have practical applications in diagnostics.

## 1. Introduction

Hepatocellular carcinoma (HCC) is the third most deadly cancer in the world [1]. Only 43% of patients are diagnosed at an early stage, and the five-year survival rate is just 31% [2]. However, most patients with early-stage liver cancer appear healthy, and show no awareness of symptoms [3]. Alpha-fetoprotein (AFP), with a molecular weight of approximately 70 kDa, is a well-known HCC biomarker [4]. The concentration of AFP is less than 10 ng mL^−1^ in healthy human serum, but increases significantly in the serum of HCC patients [5]. Detection of AFP is therefore important in the early stage diagnosis of HCC [5]. The methods most commonly used to detect AFP in HCC patients are radio- and fluorescent-immunoassays [6,7,8]. However, these methods require expensive reagents and are complicated to conduct. Hence, a simple, inexpensive, and highly sensitive detection method is required to detect AFP in the clinic.

Field-effect transistors (FETs) are promising, label-free, biosensing devices capable of detecting biomarkers [9,10,11]. FETs provide a number of potential advantages such as size, fast response, high reliability, low output impedance, portability, and low cost of mass production [12,13]. A number of studies have confirmed the high sensitivity and stability for detecting of AFP using field-effect transistors (FETs) on silicon [14,15] and electrochemical methods on carbon [16,17]. However, in these studies, AFP was diluted in PBS or purchased human serum, do not realize the detection of AFP in HCC patient plasma.

Graphene is a single atom thick, sp^2^-hybridized, carbon material. Graphene is a zero band gap semiconductor in which the conduction band and valence band are connected at the K-point. The transfer characteristics of graphene exhibit distinctive ambipolar behavior. Single-layer graphene has an extremely high carrier mobility (>20,000 cm^2^ V^−1^ s^−1^) and a large carrier concentration (~10^12^ cm^−2^) [18]. Due to its electrical properties, graphene is an ideal candidate for the fabrication of biosensors. Graphene field-effect transistors (G-FETs) have been developed to detect different types of biomarkers such as DNA, glucose, enzymes, and immunoglobulin E [19,20,21,22]. The G-FETs have been studied for the quantitative detection of various cancer markers using immunoassay methods [23,24,25]. However, the detection of AFP using G-FETs has not been reported yet.

In this study, the detection of AFP in human plasma of HCC patient was achieved using a G-FET biosensor for the first time. The G-FET was modified with 1-pyrenebutyric acid *N*-hydroxysuccinimide ester (PBASE) to allow for immobilization of an anti-AFP antibody. The structure of the immobilized antibody was observed by atomic force microscopy (AFM). The selectivity of the anti-AFP-immobilized G-FET was evaluated using human chorionic gonadotropin (hCG) and carcinoembryonic antigen (CEA). The detection of AFP was characterized in phosphate buffer solution saline (PBS) solution and human plasma from HCC patients.

## 2. Materials and Methods

### 2.1. Materials

The monoclonal anti-alpha-fetoprotein (anti-AFP), AFP, hCG, and CEA were purchased from Antibody Center (Seongnam, Korea). The human plasmas of HCC patients were provided at Keimyung University school of Medicine (Daegu, Korea). The concentration of AFP in each HCC patient plasma was verified at Keimyung University school of Medicine and HCC patient plasma were used without any purification process. PBASE and bovine serum albumin (BSA) were purchased from Sigma-Aldrich (Seoul, Korea). Large-sized graphene on a PET substrate was purchased from MCK Tech (Ansan, Korea). Ultrapure water (18.2 MΩ·cm) was used for the preparation of all solutions. PBS was made in the laboratory and was prepared using 137 mM NaCl, 8.1 mM Na_2_HPO_4_·12H_2_O, 2.7 mM KCl, and 1.5 mM KH_2_PO_4_. 0.01 × PBS (pH 7.4) was prepared by diluting 1 × PBS with ultrapure water. Antigens and antibodies were diluted in 1 × PBS.

### 2.2. Fabrication and Modification of G-FET

Gold (Au) was evaporated in a vacuum chamber (5.0 × 10^−6^ torr) to form the drain and source electrodes on the graphene sheet using a thermal evaporator and the thickness of the Au was 10 nm (Alpha-step). The gate channel size was 5 mm in width and 500 μm in length. For applying bias to the electrodes, a silver paste was used for wire bonding. Polydimethylsiloxane (PDMS) was used for the passivation of source and drain electrodes from the electrolyte. The reaction chamber was made of a 15 mL conical tube coated with PDMS. Ag/AgCl reference electrode was used as the gate electrode.

The G-FET was immersed in a mixture of 5 mM PBASE in dry dimethylformamide (DMF) solution for 2 h to achieve non-covalent modification of the channel surface, followed by rinsing with DMF [26]. After modification, a solution of anti-AFP (50 μg mL^−1^) was dropped to immobilize anti-AFP on the channel surface and it was placed in a constant temperature and humidity chamber for 9 h. Then, the anti-AFP-immobilized G-FET was washed three times with 0.01 × PBS. Non-specific adsorption was blocked by the addition of a BSA solution (1 wt %) for 60 min. Then, the anti-AFP-immobilized G-FET was washed three times with 0.01 × PBS. The anti-AFP-immobilized graphene was evaluated using X-ray photoelectron spectroscopy (XPS; monochromatic Al Kα X-ray source, 1486.6 eV, beam diameter 400 μm) and atomic force microscopy (AFM; AFM5300E, Hitachi, Tokyo, Japan).

### 2.3. Detection of AFP

The transfer characteristics of the G-FET were measured using a digital source meter (Keithley 2400, Keithley, Cleveland, OH, USA). The drain-source voltage (V_DS_) was fixed at 0.05 V and the gate-source voltage (V_GS_) was swept from 0.1 to 0.9 V. After the antigen–antibody reaction, the characteristic of the drain-source current (I_DS_) on the G-FET was evaluated in 0.01 × PBS (pH 7.4) solution. The sensitivity was evaluated by assessing the shift in voltage of the Dirac point (ΔV_Dirac_) after specific binding of the antibody to the antigen. The voltage of Dirac point was defined as the minimum current value in the I_DS_-V_GS_ characteristics of the G-FET. This study was approved by the Institutional Review Board of Keimyung University school of Medicine (IRB No. 2017-09-038-001).

## 3. Results and Discussion

### 3.1. Functionalization

**Figure 1:**
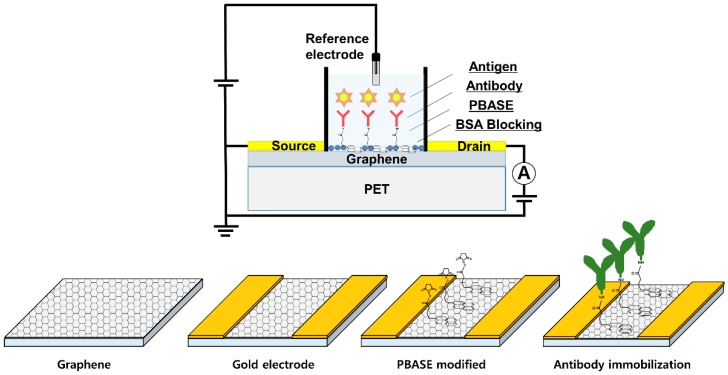
Schematic illustration of AFP detection using G-FET and modification process.

We used a non-covalent method to immobilize the antibody on the graphene surface. PBASE allows the binding of functional groups to graphene without disrupting the carbon atomic structure [27,28]. PBASE contains an aromatic pyrenyl group which physically interacts through π–π interaction with graphene sheet and a succinimidyl ester group which covalently reacts with the amino group on the anti-AFP by an amide bond [20]. A schematic diagram of the modification steps for G-FET is shown in Figure 1.

**Figure 2:**
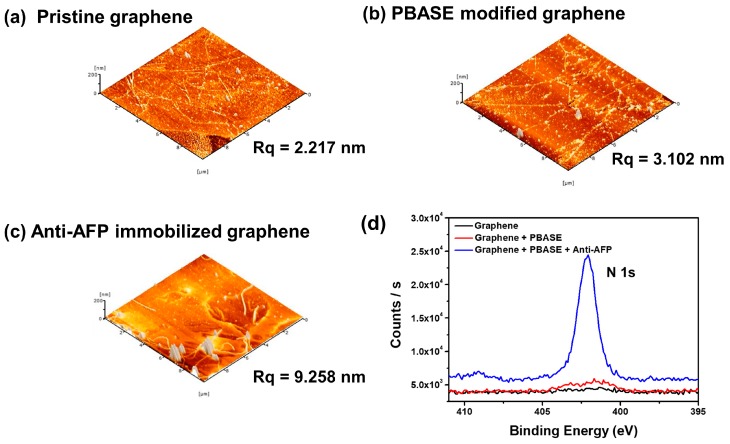
3D AFM images of (**a**) pristine graphene; (**b**) PBASE-modified graphene; (**c**) anti-AFP-immobilized graphene; (**d**) high-resolution XPS N1s spectra of pristine, PBASE-modified, and anti-AFP-immobilized graphenes.

AFM measurements were performed to examine the height of the PBASE modification and the change in the surface morphology following anti-AFP immobilization. In order to compare changes in the z-value of the PBASE-modified graphene with that of the antibody modified graphene, the roughness of substrates was evaluated at root mean square (R_q_) value. Figure 2 shows that the R_q_ value of pristine graphene was 2.22 nm. After PBASE-modification, the R_q_ value was 3.10 nm, whereas the R_q_ value of the anti-AFP-immobilized graphene increased to 9.26 nm. This increase in R_q_ value suggests that the anti-AFP antibody was successfully immobilized on the PBASE-modified surface [29]. Figure 2d shows the high-resolution XPS of the N 1s spectra on the graphene surface. After modification with PBASE, there was an increase in the characteristic N 1s peak at 402.03 eV [30,31]. After antibody immobilization on the graphene surface, the N 1s peak was significantly increased due to the presence of amine groups in the protein.

### 3.2. The Characteristic of G-FET Biosensor

**Figure 3:**
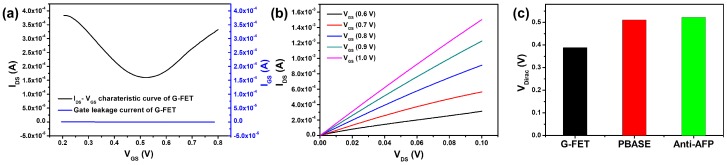
(**a**) I_DS_-V_GS_ and I_GS_-V_GS_ transfer characteristic of G-FET; (**b**) I_DS_-V_DS_ curves of G-FET; (**c**) V_Dirac_ for pristine, PBASE-modified, and anti-AFP-immobilized G-FET.

The G-FET was characterized by measuring the drain-source current (I_DS_), gate-source current (I_GS_), and gate-source voltage (V_GS_) in 0.01 × PBS (pH 7.4). The device characteristics (I_DS_-V_DS_, I_GS_-V_GS_, and I_DS_-V_GS_) of the G-FET are shown in Figure 3a,b. For I_DS_-V_GS_ and I_GS_-V_GS_, the V_DS_ was fixed at 0.05 V and V_GS_ was swept from 0.0 V to 0.9 V. The gate leakage current of G-FET was 0.3 μA. For I_DS_-V_DS_, the V_DS_ was swept from 0 V to 0.1 V and the I_DS_ increased depending on the V_GS_ in the n-channel region. The transfer and output characteristics of the G-FET in the electrolyte solution were typical of graphene FETs. The G-FET was stably worked in PBS solution without redox reaction. The intrinsic properties of the graphene did not change in an electrolyte solution. Due to the functionalization with PBASE, the Dirac point (V_Dirac_) of G-FET was shifted in the positive direction from 0.39 to 0.51 V, as shown in Figure 3c. This increase in the V_Dirac_ value suggests that PBASE with its 16 π electrons creates new scattering channels for electrons, and enhance the electronic properties of G-FET [32]. The V_Dirac_ of the anti-AFP-immobilized G-FET was shifted a further 11.2 mV, as shown in Figure 3c, because the isoelectric point (pI) of the anti-AFP antibody is less than pH 6.0 since the anti-AFP antibody is negatively charged in 0.01 × PBS (pH 7.4) [33]. The negative charge of the anti-AFP antibody accumulates the hole density on the channel of G-FET and V_Dirac_ of G-FET was increased.

### 3.3. The Detection of AFP

**Figure 4:**
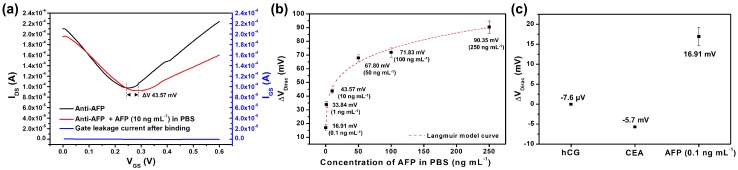
(**a**) the I_DS_-V_GS_ characteristics of G-FET before and after binding to AFP (10 ng mL^−1^ in PBS) on the anti-AFP-immobilized channel surface; (**b**) sensitivity of the anti-AFP-immobilized G-FET for AFP detection in PBS; (**c**) selectivity of the anti-AFP-immobilized G-FET for AFP compared with hCG and CEA.

The G-FET was characterized in 0.01 × PBS (pH 7.4). The pI of AFP is 4.9, and so AFP is negatively charged in 0.01 × PBS (pH 7.4) [34]. The anti-AFP-immobilized G-FET is sensitive to AFP at low ionic strengths because charge detection is most sensitive when the screening of positive counter ions is minimized in the electrolyte. The anti-AFP-immobilized G-FET was immersed in an AFP solution (10 ng mL^−1^ in 1 × PBS) for 2 h and washed three times with 0.01 × PBS. Following this, the V_Dirac_ of the anti-AFP-G-FET bound to AFP showed a positive shift of 43.57 mV as compared to the anti-AFP-G-FET alone, as shown in Figure 4a. The I_DS_-V_GS_ characteristics according to the AFP concentration in PBS were shown in Figure S1 (Supporting Information). Upon binding of the anti-AFP antibody to AFP, the hole density is increased by the enhanced negative charge. Consequently, there is a positive shift in V_Dirac_.

We approximate that each AFP binding to the anti-AFP antibody on the channel surface contains an intrinsic negative charge, which directly affects the channel surface without any neutralization by counter ions in the buffer solution. Because the Debye length of the buffer solution is 7.5 nm and the length of the anti-AFP antibody is 4 nm, the ΔV_Dirac_ depended on the concentration of AFP in PBS, as shown in Figure 4b. G-FET could detect AFP at concentrations as low as 0.1 ng mL^−1^ in PBS. When the AFP concentration was above 50 ng mL^−1^, the sensitivity of G-FET decreased, indicating that saturation had occurred. Generally, detection of AFP in the early stages of HCC patients requires the ability to detect AFP at levels below 10 ng mL^−1^. Therefore, G-FET shows the possibility as an AFP sensor. If we change the *X*-axis to logarithmic scale, the G-FET sensitivity is linear relative to the AFP concentration in PBS (Figure S2, Supporting Information).

The equilibrium dissociation constant (K_D_) for the anti-AFP and AFP interaction, with different values of V_Dirac_ at the corresponding AFP concentration, was evaluated for the anti-AFP-immobilized G-FET. The binding of AFP to the anti-AFP antibody on the graphene channel can be described by the Langmuir equation [29]:

ΔV_Dirac_ = (ΔV_Dirac,max_·C_AFP_)/(K_D_ + C_AFP_),

where ΔV_Dirac,max_ is the saturated change in V_Dirac_ and C_AFP_ is the concentration of AFP. From the fitted curve shown in Figure 4b, the dissociation constant (K_D_) was estimated to be 4.64 × 10^−11^ M. Compared with the reported binding affinity from a general characterization of the binding between AFP and the anti-AFP antibody (10^−7^–10^−9^ M) [35,36,37,38], our data indicate that the anti-AFP antibody immobilized on the G-FET biosensor has a high affinity for AFP.

The selectivity of the G-FET biosensor was assessed using other proteins, including hCG and CEA. hCG is a hormone produced by the placenta after implantation and has been found in some cancerous tumors [39]. CEA is known as a marker of cancer of the digestive system and has been shown to be increased in levels in various cancers such as colon, stomach, and pancreas, etc. [40]. The anti-AFP-immobilized G-FET was immersed in hCG or CEA solutions for 2 h and then washed three times with 0.01 × PBS. In each case, the concentration of hCG and CEA was 1 μg mL^−1^. For hCG, the ΔV_Dirac_ of the anti-AFP-immobilized G-FET was −7.6 μV, whereas the ΔV_Dirac_ of the anti-AFP-immobilized G-FET was −5.7 mV for CEA. Although the selectivity of the anti-AFP-immobilized G-FET was assessed using high concentrations of hCG and CEA, the ΔV_Dirac_ of G-FET was comparable for the AFP. These data demonstrate the practical potential of G-FET for the diagnostic detection of AFP.

**Figure 5:**
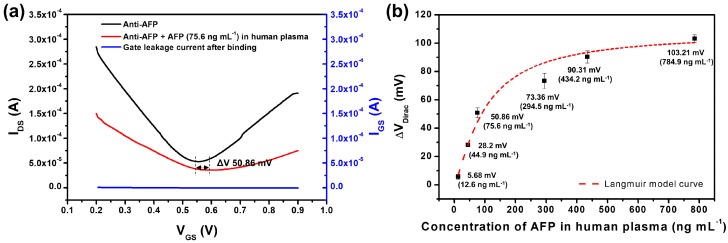
(**a**) the I_DS_-V_GS_ characteristics of G-FET before and after binding with AFP (75.6 ng mL^−1^ in human plasma of HCC patients) on the anti-AFP-immobilized channel surface; (**b**) the sensitivity of anti-AFP-immobilized G-FET for detecting AFP in human plasma from HCC patients.

The anti-AFP-immobilized G-FET biosensor was then assessed by determining its ability to detect AFP in human plasma from HCC patients. Seven samples were used to evaluate the sensitivity of G-FET, where the concentrations of AFP HCC patient plasma were 2.8, 12.6, 44.9, 75.6, 294.5, 434.2 and 784.9 ng mL^−1^. The process and conditions used for the detection of AFP in HCC patient plasma were the same as those used for the detection of AFP in PBS. However, we applied an additional washing step to remove any non-specifically adsorbed HCC patient plasma proteins from the channel surface of the anti-AFP-immobilized G-FET. The anti-AFP-immobilized G-FET was immersed in human plasma derived from HCC patients for 2 h, and then washed five times with a buffer solution. After binding had occurred, G-FET measurements were made in 0.01 × PBS (pH 7.4). The anti-AFP-immobilized G-FET was able to function in 0.01 × PBS after binding of the AFP from HCC patient plasma. The V_Dirac_ of the anti-AFP-immobilized G-FET bound to AFP showed a positive shift, as compared with the anti-AFP-immobilized G-FET alone, as shown in Figure 5a. The I_DS_-V_GS_ characteristics according to the AFP concentration in HCC patient plasma were show in Figure S3 (Supporting Information). The G-FET gave values of 5.68, 28.2, 50.86, 73.36, 90.31 and 103.21 mV at AFP concentrations of 12.6, 44.9, 75.6, 294.5, 434.2 and 784.9 ng mL^−1^ in HCC patient plasma, respectively, as shown in Figure 5b. If AFP concentration is converted into the semi-log scale, the ΔV_Dirac_ values were increased linearly depending on the concentration of AFP in HCC patients plasma, (Figure S4, Supporting Information). Unfortunately, G-FET could not detect AFP when the AFP concentration was 2.8 ng mL^−1^ in HCC patient plasma. The most likely reason for the lower AFP sensitivity in HCC patient plasma is that the anti-AFP-AFP binding interaction may have been disrupted by the additional washing process required to analyze HCC patient plasma.

## 4. Conclusions

We successfully detected AFP in human plasma from HCC patients using G-FET. PBASE was employed to functionalize the G-FET and this was successfully confirmed through XPS, and AFM. A BSA blocking process was used to avoid nonspecific interactions on the anti-AFP-immobilized G-FET biosensor. Before the detection of AFP in HCC patient plasma, a quantitative analysis of the ability to detect AFP in PBS was performed and the lowest concentration of AFP which could be detected by the anti-AFP-immobilized G-FET in PBS was found to be 0.1 ng mL^−1^. The ability to detect AFP in HCC patient plasma was assessed using six samples, with the result that the anti-AFP-immobilized G-FET could detect AFP at 12.6 ng mL^−1^. These data demonstrate that our G-FET system is able to quantitatively detect AFP in HCC patient plasma. Furthermore, antibody immobilized G-FETs have a wide potential as biosensors for early and point-of-care medical diagnosis of tumor markers.

## Supplementary Materials

The following are available online at http://www.mdpi.com/1424-8220/18/11/4032/s1. Figure S1: The I_DS_-V_GS_ characteristics of G-FET before and after binding to each concentration of AFP (0.1, 1, 10, 50, 100 and 250 ng mL^−1^) in PBS on the anti-AFP-immobilized channel surface, Figure S2: The sensitivity of the anti-AFP-immobilized G-FET for AFP detection in PBS into semi-log scale, Figure S3: The I_DS_-V_GS_ characteristics of G-FET before and after binding to each concentration of AFP (12.6, 44.9, 75.6, 294.5, 434.2, and 784.9 ng mL^−1^) in human plasma of HCC patient on the anti-AFP-immobilized channel surface, Figure S4: The sensitivity of the anti-AFP-immobilized G-FET for AFP detection in human plasma from HCC patients into semi-log scale.
