# Supplementary material for: Detection of Alpha-Fetoprotein in Hepatocellular Carcinoma Patient Plasma with Graphene Field-Effect Transistor
Source: Sensors (Basel). 2018 Nov 19;18(11):4032. doi: 10.3390/s18114032 (PMC6263997; doi:10.3390/s18114032)
Supplement: Supplementary file 1 [file sensors-18-04032-s001.pdf]

Supporting information

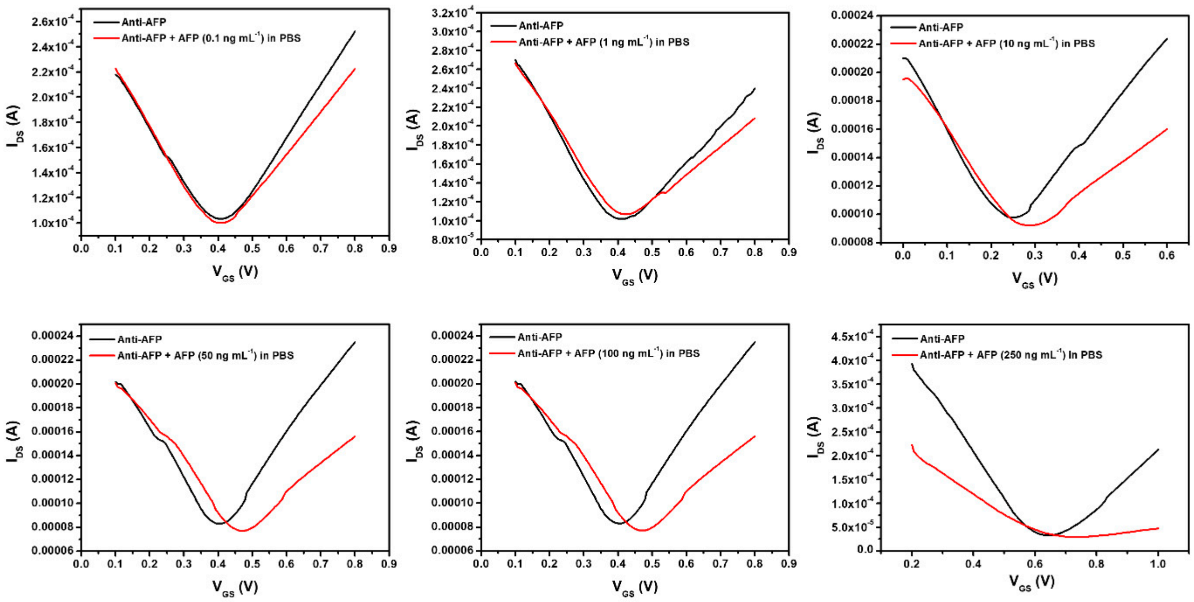

Figure S1. The I_{DS}-V_{GS} characteristics of G-FET before and after binding to each concentration of AFP (0.1, 1, 10, 50, 100 and 250 ng mL^{-1}) in PBS on the anti-AFP-immobilized channel surface.

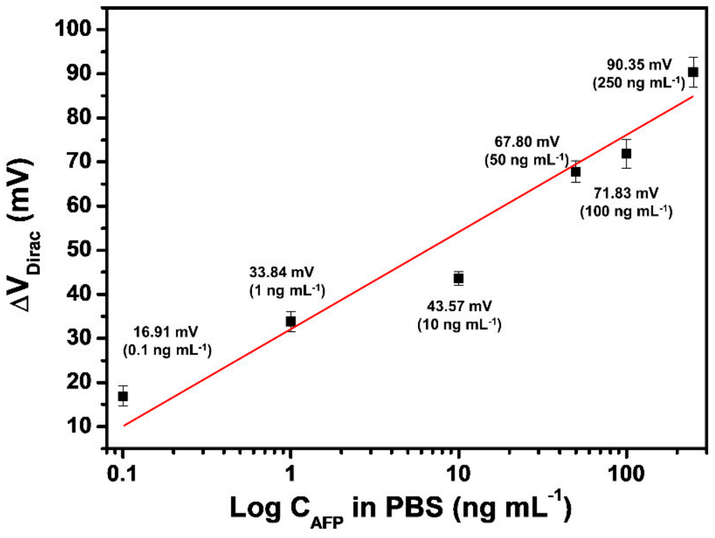

Figure S2. The sensitivity of the anti-AFP-immobilized G-FET for AFP detection in PBS into semi-log scale

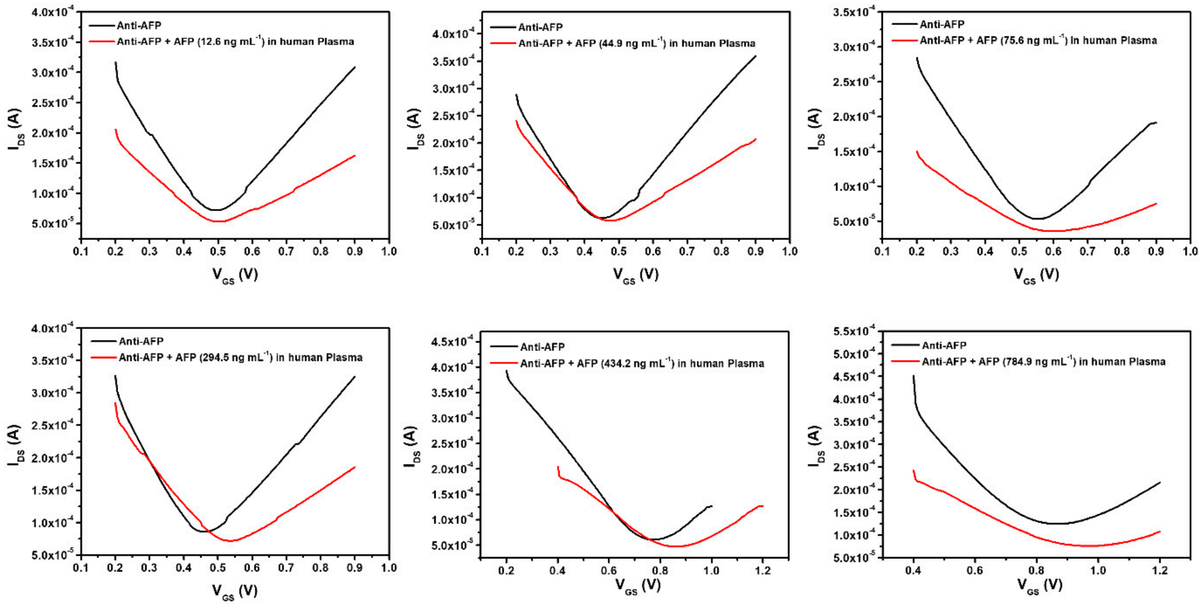

Figure S3. The I_{DS}-V_{GS} characteristics of G-FET before and after binding to each concentration of AFP (12.6, 44.9, 75.6, 294.5, 434.2, and 784.9 ng mL^{-1}) in human plasma of HCC patient on the anti-AFP-immobilized channel surface.

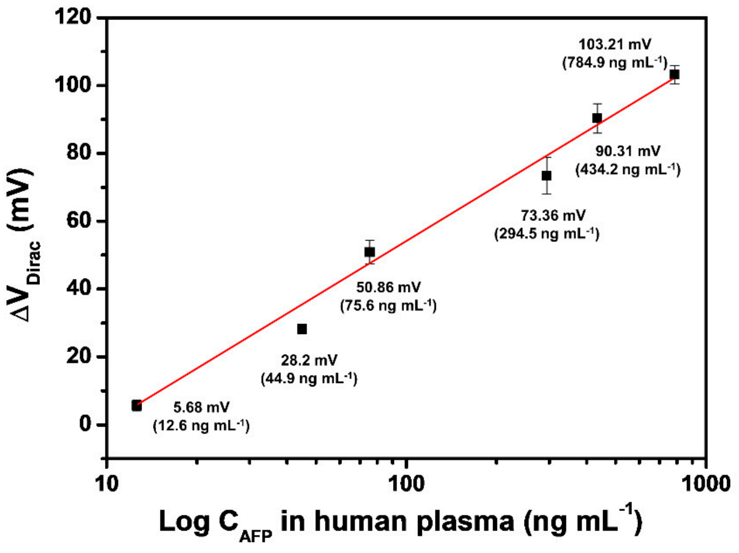

Figure S4. The sensitivity of the anti-AFP-immobilized G-FET for AFP detection in human plasma from HCC patients into semi-log scale.
